# Fictionalism of Anticipation

**DOI:** 10.1007/s12304-021-09417-z

**Published:** 2021-04-15

**Authors:** Raimundas Vidunas

**Affiliations:** Institute of Applied Mathematics, Vilnius University, Vilnius, Lithuania

**Keywords:** Anticipation, Prediction, Fictionalism, Semiosis, Complexity, Self-organization, Embodiment, Teleology

## Abstract

A promising recent approach for understanding complex phenomena is recognition of anticipatory behavior of living organisms and social organizations. The anticipatory, predictive action permits learning, novelty seeking, rich experiential existence. I argue that the established frameworks of anticipation, adaptation or learning imply overly passive roles of anticipatory agents, and that a *fictionalist* standpoint reflects the core of anticipatory behavior better than representational or future references. Cognizing beings enact not just their models of the world, but own make-believe *existential agendas* as well. Anticipators embody plausible scripts of living, and effectively assume neo-Kantian or pragmatist perspectives of cognition and action. It is instructive to see that anticipatory behavior is not without mundane or loathsome deficiencies. Appreciation of ferally fictionalist anticipation suggests an equivalence of semiosis and anticipation.

## Introduction

Human anticipation gives color to experiences and social life. Or does it even define what it means to be fully alive and engaged in the society? Trust is a form of anticipation that plays essential roles in economy, business organization, governing, politics, technological progress (Botsman 2017). For a multi-faceted example, consider the FIFA World Cup of 2018 in Russia. Various forms of anticipation are present in organization and sponsorship of the event, in the ready broadcasting industry and reliable communication technology. Prior political reservations (BBC 2014) underscore the risk of anticipation. Football itself is largely an anticipation game, especially for goalkeepers, but also for the attentive defenders, middle-field playmakers, and opportunistic forwards seeking to beat offside traps. Coaches do much anticipation work as well. And then there are expectations of football fans around the globe. At the same moment as Hirving Lozano scored a goal against Germany on June 17, 2018, seismic stations in the Mexico City registered a small earthquake (Semple and Villegas 2018). Plausibly, it was caused by jubilating fans in the city. How else can a ball kick in a Moscow stadium cause a geological event on other side of the globe, but by powers of captive anticipation?

The FIFA World Cup illustrates that anticipation is a key feature of masterly performance, better life experiences and grand scale coordination. It is indispensable for vigorous economy and functional society. An ambitious academic view is emerging that anticipation, broadly understood, is a fundamental attribute of biological life, cognition, artificial intelligence, and even of emerging, self-organizing natural phenomena beyond mechanical matter interactions. Certain universality of anticipation is noticed by Poli (2010):

“... the major surprise embedded in the theory of anticipation is that anticipation is a widespread phenomenon present in and characterizing all types of realities. Life in all its varieties is anticipatory, the brain works in an anticipatory way, the mind is obviously anticipatory, society and its structures are anticipatory, even non-living or non-biological systems can be anticipatory.”

The growing interest in broad studies of anticipation is evident (Nadin 2016; Poli 2017). Nasuto and Hayashi (2016) write:

“... anticipation is an emerging concept that can provide a bridge between both the deepest philosophical theories about the nature of life and cognition and the empirical biological and cognitive sciences steeped in reductionist and Newtonian conceptions of causality.”

According to Nadin (2016: 283), anticipation is “a definitory characteristic of the living”. This echoes Rosen’s (1985) distinction between simple, mechanical systems and complex, living systems. Similarly, *predictive coding* (Clark 2013; Pezzulo et al. 2018) and *active inference* (Friston et al. 2016) are key features of cognitive and biological processes in their free energy formalization (Friston and Stephan 2007; Ramstead et al. 2018).

Working definitions of anticipation in academic literature (Poli 2017: Ch. 1) refer either to future prediction (Poli 2010), or to representation of self and the environment (Rosen 1985). These definitions do not mention *fictionalist* aspects as a conspicuous feature of anticipation. According to the linguistic definition (Matti 2019), *fictionalism* accepts statements of a discourse not as literal truth but as useful fiction of some sort. Similarly, I see anticipatory cognition as having a pragmatic heuristic rather than rigidly representational character, and as generally resilient to possible and inevitable errors. As an alternative condition to belief or disbelief, anticipation is compellingly understandable in fictionalist terms.

This article constitutes a primer introduction to the overlooked fictionalist facets of anticipation and their deep going implications. It is worth mentioning that *fictional expectations* in economics are accentuated by Beckert (2013). The fictional character of anticipation is demonstrated amply by the current COVID-19 pandemics that causes huge disruptions in the global economy, travel, sports events, and thereby reveals the regular expectations as fictitious plans at heart. Ingrained routines became unsettled or counterproductive. Bryant’s (2020) early philosophical essay on the pandemics is a good accompaniment to this article.

I highlight two fictionalist aspects of anticipation that appear to counter the leading contemporary paradigm of cognition based on *predictive coding* (Friston et al. 2016). Firstly, anticipatory action includes not only exciting possibilities of learning, novelty seeking, rich experiential existence, but also mundane or even repellent facets such as prejudiced behavior and stressful reactiveness. If human judgment can be patently biased, fallible and irrational (Kahneman 2011), more primitive forms of anticipation can be expected to be even more superficial, fallacious, crude. With a contrasting reference to *behavioral economics* (Minton and Kahle 2014), the ambitious thesis of predictive coding that cognitive and living systems are effective probabilistic prediction machines is comparable to *rational choice theory* (Gilboa 2010).

Secondly, I argue that the established frameworks of anticipation, prediction, autonomy still under-appreciate active, generative drives whereby anticipating beings seek to fulfill or impose their *existential agendas*. The frameworks of representation, predictive coding, and *autopoiesis* (Maturana and Varela 1980) portray a reactive, stasis-oriented manner of observation, learning and adaptation. Even the time-centered approach (Poli 2010) has a flavor of reactiveness to future. But anticipation can be spatial as well, as in venturing to new locations or encountering new objects. New experiences and exploits are often attained by own new behaviors, improvised persistence. Complementarily to the approach of *enactive embodiment* (Varela et al. 1991) of the environment, cognizing anticipators effectively seek to enact their destined actions in the world.

The next section reappraises the scope of observed anticipatory behavior, including mundane or loathsome manifestations. Section “Philosophical Parallels” defines the emergent *fictionalist stance* of anticipators, and finds similitude in several philosophical currents, particularly in the Kantian synthetic a priori categorization and American pragmatism. Vaihinger’s “The Philosophy of As If” (Vaihinger 1935) and Santayana’s “Scepticism and Animal Faith” (Santayana 1955) match well with the anticipatory fictionalism in complementary ways. Section “Existential Agenda, Feral Anticipation” gives key definitions of *anticipatory plots*, *existential agendas*, discusses formalization of anticipation itself, and touches on causal powers of *feral* anticipation. Section “Logistics and Mythology” contrasts entrenched, dependable plots of functional anticipation with indefinite, uncertain scripts. This localizes applicability of the stronger mythological language. Section “Embodiment and Semiosis” explicates embodiment and semiotic unfolding of anticipations and existential agendas. The last section underscores broad significance of fictionalism.

## The Scope of Anticipation

Fragility and forcefulness of being alive constitute a subtle polarity. On the one hand, the environment is ever changing and rudimentarily unpredictable. There is no certainty that an acorn will turn into an oak tree. At best, an acorn effectively *anticipates* favorable conditions for appropriate employment of its nutty nutrients and DNA guidance. Even animals have objectively limited control over own fates. Some of their maturation phases — such as winning a duel for status, finding a sexual partner — are only roughly determined by the fixed biochemical mechanisms or scenarios. The whole trajectory of the Aristotelian *telos* of a living being depends on many things going right, sometimes sporadically and extraordinarily right. In a sense, an organism lives in anticipation of favorable luck and certain outside help.

On the other hand, organisms act powerfully on the environment. Fulfillment of anticipation is followed by resolute activity that intervenes in the ambient dynamics of the environment and own organic development. In aggregate, the biosphere changes the geology and the atmosphere of the Earth.

Representational models of anticipation capture this polar dynamics poorly. Rosen (1985: §6.1) defined an *anticipatory system* as a natural system that contains an internal predictive model of itself and of its environment, which allows it to change state at an instant in accord with the model’s predictions pertaining to a later instant. This presupposes significant cognitive capacities that normally require a brain. The advance from prediction to action *at an instant* is not clear; say, how does a predicted scenario lead to a decision when the scenario is unfavorable? Rosen’s formal structure of anticipatory modeling is particularly inapplicable to the animal behavior in predator-prey races, where the action is very fast, hardly predictable, contingent on accidental features of the environment, and the outcome is uncertain. Organisms cannot have a comprehensive model of the environment and its possible changes. Instead, an organism works from its *Umwelt* (von Uexküll 1957; Kull 2010), i.e., its functionalist-semiotic view of the environment (and itself). A living being filters the perceived environment for existential necessities, threats and *affordances* (Gibson 1966). Action is triggered by rather few cues out of a mass of environmental information. For an example, consider seasonal phenological cycles (Schwartz 2003; Forrest and Miller-Rushing 2010), particularly the spring revival. They constitute webs of anticipatory attentions, responses and influences without any organism apprehending wholly its environs.

To appreciate the scope of anticipation, we should recognize it in mundane, commonly failing, or even loathsome forms as well. Examples in human social contexts are: stereotypes, prejudice, superstition, strong first impressions, adoration of leaders. These anticipations determine human behavior to a larger extent than rational thinking. Comparable anticipations in the biological world are checked perhaps only by natural selection. A different example is the physiological stress response (Sapolsky 1994). For most animals, it is an episodic anticipatory reaction to adverse environmental conditions. But it is chronically triggered in the modern human life with harmful effects on health.

On the other hand, higher levels of existence beyond being mere matter require determined anticipation, in a sense. Just being alive is inherently an anticipation of further favorable conditions. Anticipation or being anticipated can define agency (Poli and Valerio 2019; Simondon 1964). Anticipators act elementally from anticipatory *fictions* rather than from *representations* of future or the world. Workable fictions are often *reflexive* (Bourdieu and Wacquant 1992): they “represent” worlds that would not exist without following those fictions, including reflexively anticipated worlds that do not exist yet and may never exist. The fictional character of anticipation is particularly notable in carrying through long-term purposes of existence.

Own action is the critical reflexive element. Its productive effect is characterized much more elegantly as anticipatory fiction rather than representation. An example of elaborate action with far-reaching reflexive expectancies is *niche construction* (Laland et al. 2019), exemplified by dam building by beavers, or soil modification by earthworms (Nuutinen 2011). Niche construction is supposed to improve the quality of the environment for the offspring, also under the ensuing long-term ecological dynamics.

I argue that a worldly cognitive being does more than playing “the game of predicting the sensorium” (Allen and Friston 2018: 2464). It has an *existential agenda* delineated by its *anticipatory plots*, as I define later. The fictions have variable significance and probability of actualizing. For a while here, I start testing mythological language in its both delusional and generative or stimulating meanings to underscore these variabilities.

The penetrative contrast between observing and active anticipation is well captured by the famous quip of Marx (1845: Thesis 11): “The philosophers have only interpreted the world in various ways; the point, however, is to change it.” Ironically, the prototypical examples of consequential impetuous change happen to be capitalists like John D. Rockefeller. The *modus operandi* of entrepreneurs is brazenly mythological rather than analytical. Their innovative action is formed by incomplete visions, ambitious anticipations, and quickly devised plans. For example, Rockefeller’s success was furthered by his determined, optimistic appraisal of the risks in the early oil industry (Chernow 1998: Ch. 6, 16). He daringly expanded his oil business in an unstable market, despite uncertainty of how much oil would ever be yielded from the Pennsylvania fields or anywhere else. He entreated partners to hold onto Standard Oil shares, or willingly bought them from disgruntled stockholders (Chernow 1998: 168, 181, 380).

Entrepreneurs rely on their experience largely in a mythological mode as well; high rates of venture failure attest to that. Crises are commonly resolved by essentially betting on a fortunate strategy. For example, the diverging fortunes of Kodak and Fujifilm — the two largest manufacturers of photo films until the 2000s — are attributed to different decisions in coping with the swift competition of digital photography (Kmia 2018). Fujifilm wagered on massive production of LCD screens, even if the competition from the plasma technology was intimidating.

I argue that the anticipatory aspect of aspirational mythology deeply unifies human sciences with biology, ecology, and eventually with self-organizing phenomena in general. Living or complex existing forms require specific dispositions, habits (Fernández 2012), systemic-communal “practices” and established interaction patterns for effectual adherence to own survival interests. These associations tempt toward anthropomorphic generalizations and *pansemiotics* (Salthe 2012). While making a similar argument, Ulanowicz (2010) quotes Bertrand Russell (1960: Ch. II):

“Every living thing is a sort of imperialist, seeking to transform as much as possible of its environment into itself and its seed. [...] We may regard the whole of evolution as flowing from this ‘chemical imperialism’ of living matter.”

More benign but similarly active aspects of human experience and learning are underscored by Dewey (1916: Ch. II, XI). The *direction-to-fit* distinction (Searle 2001: 37–38) between beliefs (as having to fit the world) and desires (as seeking to alter the world) is a kindred philosophical discussion. Let us take a look at other philosophical confirmations.

## Philosophical Parallels

Western philosophy has been in opposition to mythological interpretation of the world since the Greeks (Robinson 2004: Lect. 2). Modernist philosophy, especially positivism (Ayer 1936), has yet greater distaste for speculative, metaphysical narratives. But reversal of Comte’s (Comte and Lenzer 1975) theological-metaphysical-positive historical progression of knowledge is worthwhile to consider when formulating a primitive epistemology for simpler living or cognizing beings. A good reference point is MacIntyre’s (1981: Ch. 10) view of the ancient societies, where everyone had to know own place in the community as well as correspondent privileges, duties, performance norms; where courage, loyalty determined reliance for friendship, et cetera.

My proposal boils down to assigning a pragmatic *fictionalist* (Matti 2019) and *fallibilist* stance to cognizing, anticipating beings towards future, own capacities and fate, and the indirectly apprehended environment. They are corporeally ready to employ their developmental stories as useful, even vital fictions rather than comprehensive, unambiguous verities. As I discuss here, indirect support for viability of the fictionalist stance can be found in philosophy of science and post-modernist ideas. The stance embraces the Kantian a priori categorization and American pragmatism liberally. The fictionalist stance is anti-realist epistemically, but onticity of reality is acknowledged implicitly: there would be no set out fiction without the opposition to reality.

Fulfillment of aspirational expectations is never guaranteed. But rational, empirical or post-modern skepticism (Popkin 2003) leads to the conclusion that any anticipation, knowledge or conviction are open to failure. According to anti-realist currents (McCain 2016; Massimi and McCoy 2019), scientific knowledge differs only in commitment to reliability and technical standards as a set of predictions and extrapolation of perceptions. Popper (1962: 66) writes: “Science must begin with myths, and with the criticism of myths.” Living out anticipatory myths is similarly inescapable as falsification of scientific theories. Biological cognition and anticipation are probably closer to superstition, faith than to the best scientific practices such as *Bayesian inference* (Knill and Pouget 2004). Rather than focusing on a few well-defined, immediate problems of life, the organisms may inherently follow reflexive behavioral myths that encompass necessary wisdom for their whole term of existence. Downsides of a priori beliefs and anticipatory organization can be mild, while probable rewards could be existentially enormous, like in Pascal’s wager (Hájek 2018). From the skeptical perspective, life is an art of being right for wrong reasons. Or in other words, the organisms rely substantially on *epistemic luck* (Pritchard 2005), particularly when making fight-or-flight, migration or mating decisions.

The relation between aspirational fiction and life is reminiscent of *psycho-physical parallelism* (Walker 1911), particularly of the Spinozian notion that mental and physical events do not interact causally, but are coordinated as two attributes of God. In our context, the fictions and the physical reality are coordinated by a generalized natural selection. Thereby emergent mythological meaning defines the teleology of the being and intentionality of its behaviors. The extent of the parallelism can be extraordinary: the DNA guides the development and the living of organisms within viable contexts; values of individuals or societies direct their fate and history. Operative myths constitute the semiotic DNA of the being, a critical causal factor of its ways.

Extending Kant’s (1998) transcendental turn, the myths can be seen as the synthetic a priori knowledge of the cognizing being. They dynamically organize and mold its perception (and action!), impose “intuitive” frames of apprehension, stabilize experience and performance. Anticipation itself is a kind of categorization of future scenarios. Fictional expectations as assorted Kantian-like categories determine the *Umwelt* (von Uexküll 1957) and routine perceptions of the cognizing being. Vaihinger’s (1935: III.A) interpretation of Kant’s *ideas of pure reason* as self-conscious fictions with practical benefits grounds his philosophy of As If. Vaihinger (1935: III.D) credits Nietzsche with alike association of neo-Kantian ideas of instrumental cognition with Darwin’s natural selection. In the same vein, evolutionary epistemology (Lorenz 1977) affirms that the synthetic a priori knowledge is shaped by natural selection. This implies that workable semantics and competences appear first in partly ad hoc ways. The world is thereby a natural selection of myths.

The fictionalist perspective matches well with subtleties of post-modernism. One point of agreement is that all cognition is inferential and mediated by signs (Cahoone 2010: Lect. 31). Variable slicing by different perceptions and categorizations naturally leads to perspectivism. Derrida’s (1974) critique of Western logocentrism is conforming here, but his radical *deconstruction* is antithetical to appreciation of myths. Eventually though, a workable myth is to be understood roughly uniquely. Brashly rephrasing Foucault (1980), *mythology is power* — no less potent as organizing or generative power than possibly oppressive. Contrarian and pluralistic confirmations can be found in Lyotard’s (1983) critique of metanarratives, and his account of the post-modern abundance of *little narratives, language games.*

Not least, the outlined fictionalist stance matches well with American pragmatism (Legg and Hookway 2019), particularly with:

- Peirce’s (Peirce et al. 1935: 1.141) *fallibilism*; i.e., the epistemological view that no belief or theory can ever be certain;
- anti-skepticism (Putnam and Conant 1994: Ch. 8);
- Peirce’s inquiring logic of *abduction* and speculative grammar (Fann 1970; Ejsing 2007; Bellucci 2018);
- James’ (1896) *will to believe* as the necessary practical will for required, purposeful action and fulfilling experience;
- James’ *functionalist*, purpose-driven psychology (Robinson 2004: Lect. 47).

Peirce (1935: 1.545) replaced Kant’s preformed categories of understanding and forms of intuition by a dynamical stock of signs (Cahoone 2010: Lect. 17). Just as Peirce’s (Peirce et al. 1935: 5.283) implicit theory of mind postulates that all thoughts are signs, biosemiotics (Emmeche and Kull 2011) proposes that animal perception, communication, behavior and metabolism are ubiquitously mediated by signs. Anticipation within systems is recognized as a semiotic process by Kull (1998) and Nadin (2012). Individual anticipation can be bluntly seen as a Peircian triadic sign (Savan 1988): a cause to anticipate can be viewed as a *signifier* (i.e., representamen), fulfillment of the anticipation as the correspondent *signified* (i.e., object), and the consequential process or its supposed scenario as the *interpretant*. Own action of the anticipator is typically a crucial part of the interpretant process of converting a signifying affordance to a welcome consequence. Accordingly, anticipators or their *habits* (West and Anderson 2016) could be considered as general manifestations of Peirce’s thirdness.

The difference between pragmatism and Vaihinger’s (1935: viii) fictionalism is that the latter admits theoretical falsity of usable ideas, while pragmatism ties fruitful ideas to the definitions of truth and knowledge. I lean to the pragmatist side in seeing reflections of reality in workable notions and dispositions. Santayana’s (1955) naturalism is even more to the point. It postulates *animal faith* of vital, ingrained beliefs that are essential for action and cognition. Continuing the pragmatist gist, Rorty (1979) denied foundational justification of knowledge and definability of truth. He affirmed Davidson’s (2001) veridicality of existing beliefs. The truth of (mythological) knowledge could be established by the depth and the temporal extent of the parallelism with the surrounding reality and, pragmatically, with own existential purposes. The parallelism can be limited by environmental change, ecological or parasitic invasion, or own unsustainable influence.

## Existential Agenda, Feral Anticipation

Broad universality of anticipation invites recognition of anticipatory capacities, teleological agendas in simplest cognizing, self-organizing beings. Contrary to (Rosen 1985; Nadin 2012), I consider perception-reaction cycles as prototypical anticipating entities already. *Primed dynamical systems* of (Vidunas 2019) can be recognized as *radically open* (Chu 2011), critically sensitive, provoking and causation delegating anticipators. Here I give resonating definitions of *anticipatory plots*, *existential agendas*, and discuss briefly formalization of anticipation itself. Instead of affirming the coextensiveness of semiosis and life in biosemiotics (Sebeok 2001), I suggest an equivalence of semiosis and anticipation. In addition, I formulate the causal power of *feral* anticipation.

An *anticipatory plot* is a sequence of anticipations, responding actions, set outcomes, and further anticipations, actions of a cognizing being. It is an implicit script of what *could* happen given the right context. The script does not have to be rigid or definite, but may be approximate or flexible, and may have relative gaps to be filled in opportunistically. Anticipatory plots should match cognitive capabilities of the anticipator; excitatory (though not necessarily productive) reaction to anticipation fulfillments has to be possible or probable. The prescribed reaction may be objectively possible only under extraordinary circumstances, or with some “magic” assistance not specified by the anticipation. For example, an elephant *might* fly steadily under exceptional stormy conditions, possibly filling in a plot gap thereby. In the next section, I differentiate anticipatory plots by their plausibility or routine reliability, and suggest mythological terminology for the less dependable yet vital anticipated scenarios. Anticipatory plots address autonomy, subsistence and relational organization of the anticipator. Interesting plots are those enhancing quality or probability of prolonged existence of the anticipator.

An *existential agenda* is a set of anticipatory plots of a cognizing being, together with their semantic meaning to its existence. It is a set of implicit anticipations, adumbration of what *should* happen. For example, a stray cat seeking an owner has an existential agenda, with several behavioral scripts to attract her or him. Biological *life* can be defined as an existential agenda that includes metabolism, self-repair, and reproduction. Emergence and evolution of life could be described within a spectrum of existential agendas. This spectrum can be imagined starting with Maslow’s (1943) hierarchy of human needs by extrapolating it to existential agendas of mammals, vertebrates, multicellular and unicellular organisms, and eventually to virtually biotic hypercycles of chemical reactions. Existential needs will vary across the food chain, within territorial or hierarchical species, down to parasitic organisms, and so on. The variable complexity of agendas allows variable complexity of requisite biochemistry and information processing. Graves’ (1970) *levels of existence* follow Maslow’s hierarchy to a great extent, and fit into the delineated spectrum of existential agendas even better.

A technical definition of anticipation itself is perhaps premature, because usage of this notion shifts with newly appreciated limitations of representational models and future prediction. Radical openness of anticipation is well characterized by Deacon’s (2011: 27) *ententionality*; he uses the term *ententional* as “a generic adjective to describe all phenomena that are intrinsically incomplete in the sense of being in relationship to, constituted by, or organized to achieve something non-intrinsic”. Cryptically, ententionality encompasses self-preservation, adaptation, functionality, satisfaction conditions, purposes, subjective experiences (Logan 2012) — in a word, anticipation. The primary aspect in my focus is structural readiness for favorable conditions and predisposed self-enhancing reactions, behaviors or dynamics. That readiness constitutes a whole anticipatory story. *Delegated causality* in (Vidunas 2019) stipulates structural readiness for external perturbation, but the positive value of the ensuing interaction may be missing. We would not say that humanity *anticipated* the COVID-19 pandemics with its unpreparedness and institutional vulnerability.

Contemporary biosemiotics postulates that life and semiosis are coextensive (Sebeok 2001), as both are teleological processes of functional organization (Kull et al. 2009). I rather suggest that semiotic processes are coextensive with broadly understood anticipation. As mentioned in Section “Philosophical Parallels”, anticipation is a semiotic process on systemic (Kull 1998; Nadin 2012) and participatory individual levels, and even a Peircian triadic sign. An anticipator is a signifier of being alive or of the signified performance, opportunity, while own action, environmental processes, energetic particles are the interpretants. Anticipatory readiness points to a future scenario; thereby it performs a semiotic indication and is teleological. Anticipation pertaining to own action is tantamount to *intention.* The transpiring functional scripts and existential agendas have a holistic character, like good literary fiction.

The association of anticipation with semiosis, recognition of anticipatory behavior in simple dynamical structures, and fictionalist construal of potential meaning are likely to lead to definition of very low *semiotic thresholds* (Rodríguez Higuera and Kull 2017). The *protosemiotic* (Sharov and Vehkavaara 2014) threshold of associating signs with action (“know-how”) rather than with objects (“know-what”) can be met by the mentioned primed dynamical systems already. Fernández (2015) highlights “appropriate receptive structures” in the interplay of semiotic and physical regulation. Effective anticipation of the receptive structures constitutes a dual causal force to the developed top-down regulation.

*Speculative realism* (Harman 2002; Bryant 2020) articulates the reality that capacities of existing objects are inexhaustible by cognitive schemes of observers and consumers, or wilderness of *feral things* (James 2019). In contrast, I propose that complexity and life arise prototypically from interactions of *feral anticipators*, that is, from feral categorization, association or semiotics.

## Logistics and Mythology

Working representation of anticipatory plots or enaction of existential agendas require material embodiment and a whole logistical system of furnishing essentials. Anticipatory plots are fulfilled by *following* them by means of dispositions, habits, learned behaviors, recognition of the expected context, referral to information carriers. They identify systemic (or ecological, social) constraints and familiar patterns as signs. For example, consider the DNA molecule that constitutes basically an embodied mythological story of the development and the living of an organism. It gives the “words” to the anticipating biochemistry. The reflexive machinery with ribosomes, RNA polymerase, transfer RNA (Berg et al. 2006) exemplifies existential, material modalities of the biochemical mythology.

Incidentally, is the language of mythology justified right here? On the one hand, biochemical functionality and organic development are amazing and still mysterious in their arrangements. They are mythical in the nihilistic sense as well, since so many physical interventions may wreck the fine biological organization. On the other hand, routine biological meanings have to be taken at face value in organic employment or investigation. Numerous instances of optimized biochemical or physiological functionality (Bialek 2012) constitute a firm basis for organic behaviors and their biosemiotic interpretation. Mythological vocabulary should rather not be used beyond initial rhetorics to characterize entrenched, dependable functionality.

Still, the current point is that biochemical fictions of normative organic functionality require a lot of logistical support. Besides genetic guidance, resourceful systems rely on nutrient supply, waste removal, homeostasis, neural and hormonal coordination on various scales. The right contexts and logistical support are parts of anticipatory plots. Many vital physiological mechanisms are structurally deeply protected from surprises. Organic health and well-being depend on orderly actualization of developmental plots and regular anticipations.

Importance of the functional logistics is acknowledged by *constructor theory* (Deutsch 2013; Marletto 2015). For any physically possible circumstance or transformation, constructor theory postulates existence of a *constructor*, that is, an object or a process that can repeatedly and reliably bring that circumstance about. Like relational biology (Rosen 1985) or the notion of *autopoiesis* (Maturana and Varela 1980), constructor theory focuses on abstract organizational requirements and processes. The organizational relations have an anticipatory character, really: each involved substance fills in an expected requisite role, and more importantly, the material substances are radically open to particular demanded interventions or informational guidance.

Reliability of designated functional mechanisms is variable. *Allostatic* (Sterling 2012) regulation through anticipatory change of somatic parameters is less firmly reliable than homeostasis. The neural-cognitive control of behavior is no less prone to errors. Here probabilistic models of *predictive coding* (Friston et al. 2016) apply most fittingly. Further, the genetic script for the whole lifespan may contain gaps, that is, relatively much less specified scripts for developmental or living events. In particular, sexual mating may “purposely” have indefinite, open-ended facets that would, for example, channel environmental conditions and stabilize natural selection. Lifetime learning may evolve not only through cognitive capacities, but also through anticipated patterns of growth, trials and lifetime semiosis. The comprehensive lifespan script may include a *habit change*, entailing a messy cognitive overhaul. Campbell’s (1968) *monomyth* of Hero’s Journey could be a good guidance to archetypical metamorphoses that are subtly anticipated in biological-cognitive lives. It is for these underspecified, barely probable scenarios that mythological language would be appropriate.

Anticipatory plots do not have to be restricted to learning from past experiences or resemblances. They may encompass merely feasible but bold existential agendas, and some implicit wisdom regarding *unknown unknowns* (Logan 2009). Less definite but gradually effective semiotics should be particularly characteristic of ecological interactions (Ulanowicz 2010). Synergetic mutualisms arise from congruous anticipations whose actualization is somehow protected. Interactive categorizations can have a flavor of socio-cultural framing (Cassirer 1953).

Both evolution and a single life prompt action in learning environments of *low validity* (Kahneman 2011: Part III). The list of human cognitive biases, fallacies, and heuristics (Kahneman 2011) should be a good guide of how spontaneous or anticipated semiosis happens routinely — even if common failures to employ more objective means of cognition would remain to be explained. Particularly interesting are the cognitive biases based on story formation: the narrative fallacy, the halo effect, valuing associative or causal coherence. The propensity to story development reflects key importance of anticipatory plots in any evolving cognitive-semiotic system, I reckon. The operative stories could be analyzed using the multivalued semiotics of Greimas’ (1987: Ch. 6–8) *narrative grammar*.

From the perspective of Peircian semiotics, representational cognition and predictive learning rely on indexical signs mainly. *Code biology* (Barbieri 2015) describes the well-established, reliable semiotics of homeostasis, development and reproduction. It is profuse with indexical signs as well. The less specified semiotics of fairly opportunistic living requires association and interpretation. *Biohermeneutic* approaches (Markoš 2002; Chebanov 1999) are applicable then. The emergent interpretation ought to aim at fitting the existential agenda of the organism. “Creative” association of triadic symbols generates varied anticipatory plots that could meet viable contingencies productively.

## Embodiment and Semiosis

How does semiosis develop, either spontaneously or by inherited anticipation? The focus should be on employment of already available material and cognitive resources, or *semiotic scaffolding* (Hoffmeyer 2015). Anticipatory relations can buildup innately bottom-up, starting from primed structured materials and their “idealistic” demand for particular interventions. That demand is normally satisfied eventually by distinct substances. The whole vehicle of living relations is reconstructed in a born organism as a “free market” of primed genes and proteins. Available and emergent signs are linked on various scales into hypothetical patterns whose experiential affirmation is anticipated. Less reliable signs and awaited coincidences fit sporadically but productively into anticipatory plots and existential agendas.

Emerging demands of the functional organization can be satisfied only by present substances, which are likely to have unrelated other roles or original conditions of existence. The substances become new *affordances* (Gibson 1966) for the most open-endedly anticipating components. This dynamics constitutes a form of *embodiment* (Glenberg 2010) and *semiotic scaffolding* (Hoffmeyer 2015). For example, biological information careers probably evolved as successful targets of guidance “requests” from the anticipators, starting from arbitrary, “superstitious” sensitivities of the anticipators. This fits the paradigm of *extended cognition* (Clark and Chalmers 1999), epitomized by the behavior of consulting a map or a notebook.

A general mechanism of embodied fulfillment of anticipatory inquiries could be quick organic development of rich motor repertoire and mannerisms by referring to loosely related experiential memory. With that, possibly fitting knowledge is transferred across physical modalities or scales by expedient analogy. The transplanted information can be most completely encoded in one perceptual-motor modality in a manner insinuated by the theory of visual, auditory or kinesthetic *learning styles* (Pashler et al. 2008). These *virtual* embodiments are based on cognitive rather than physical resources. In that vein, behavioral economics (Kahneman and Tversky 1984) describes how human choices are determined primarily by emotional or contingent *framing* rather than objective merits of the choices. Likewise, momentary animal interpretations and decisions are spontaneously generated based on contingent clues, impulses or impressions, without anything like objective deliberation generally. Embodiments arise as *spandrels* (Gould and Lewontin 1978) rather than adaptations: they are incidental scaffolds for emerging new capacities and substantive purposes.

Semiosis translates recognized resources and dynamic processes into expected utility under my view that semiosis and anticipation are coextensive. Affordances and recurrent sequences of events become Peircian signs, whereby initial perceptions or triggers signify eventual benefits or outcomes under “interpretant” action or dynamics. The meaning of the signs is pragmatically fictionalist rather than precise, logocentric. Bounds of the *recursive semiosis* (Peirce et al. 1935: 1.339) — presumably, toward fundamental physical interactions in one direction, and some cosmic selection in the other — are disregarded by the fictionalist stance of anticipators, as their operative level of interpretation ignores dynamical details, thermodynamic limitations, higher meanings. The most reliable signs establish persistent patterns of behavior and experience. They provide the embodiment frame for semiotic scaffolding towards rich functionality and interaction. Less reliable signs are the focus of emergent creative manipulation by a kind of free association; they become leverage points for flexible adjustment, learning and communication. Systemic or communal tendencies may evolve for stabilizing precedents and “customs”.

Semiotic scaffolding may recursively continue beyond material embodiment. This virtual embodiment across cognitive levels can be recognized in the techniques of competitive memorization through rich association or navigation scenarios (Foer 2011; O’Connor 2019), and in abstract cognition through metaphorical bodily sensations (Carpenter 2011; Sapolsky 2017: Ch. 15). An example of the latter is moral disgust registered as physical disgust. The James-Lange theory (James 1884) that emotions are initiated physiologically rather than mentally is another exemplar of embodiment dynamics. With genuine emotions, the *somatic markers* (Damasio 1994: Ch. 8) imitate Hebb’s (1949) dictum “Neurons that fire together wire together” and fire together with the processing brain circuits. These scaffolded signals have great weight in decision making, evidently.

Focusing on the “free market” aspect of the semiotic interaction between anticipators, I recapitulate as follows. The demands of existential agendas are satisfied by haphazard, opportunistic embodiments of affording services in various forms of material modalities and cognitive constructs. This interaction of bio-economic *demand and supply* should extrapolate to anticipatory capacities and teleological agendas of simplest cognizing, self-organizing beings. The simplest *Umwelt*, existential agenda or Peircian habit of a primed dynamical system can be recognized in mere organization of the particular reaction. Anticipators constitute (generally non-neural) *dispositional representations* (Damasio 1994: 102) of demands and opportunities in the environment. The existential agendas of many entities may include becoming effectively well-designed, *strangely familiar* (Botsman 2017: Ch. 3) affordances to others, or fitting competitively into *centripetal* (Ulanowicz 2009: Fig. 4.3) autocatalytic flows. These emergent drives are analogous to the objectives of the design industry (Hinton 2014: Ch. 4).

## More Fictionalism

Recognition of anticipatory behavior in complex self-organizing phenomena has massive interpretive power. In turn, the fictionalist facets of anticipation clarify normativity, holism, teleology, striving of living or cognizing beings, and untangle conceptual complications of malfunction, excess and disequilibrium. Kindred anticipatory notions of *Umwelt* (von Uexküll 1957), affordances (Gibson 1966), functionality (Ariew et al. 2002), abilities (Maier 2018), dispositions (Choi and Fara 2018) can be similarly smoothly analyzed from the fictionalist perspective. Norms, meanings, intentions, goals, beliefs, signals are fictions whose proper unfolding can be usefully anticipated. As the poet Muriel Rukeyse (1968: IX) writes: “The Universe is made of stories, not of atoms.”

Semiotics and even philosophy of language could embrace the fictionalist approach rather than the customary logocentric setting. Adopting the spirit of Vaihinger’s (1935) expedient illusion, the meaning of a sign or an utterance becomes a fiction that has to be construed well by the listeners or the interpretants. Processes of communication and learning encompass homologous fictions of proper comprehension. Davidson (2005, Ch. 6) describes these fictions in communication as *passable theories*. Even conventions are likewise anticipatory, thus fictional, tools for minimizing misunderstanding. As well, confidence in the meaning of words and signs can be compared to Santayana’s (1955) compulsive *animal faith*. In all, my proposal constitutes a strong kind of *hermeneutic fictionalism* (Woodbridge and Armour-Garb 2010) towards the context of communication and the meaning of used language.

Fictionalism can be applied to theory of mind (Demeter 2013) to the extent that other mind is as unknown as the future or a novel environment. Knowing the unknown in the messy, competitive world can be accomplished opportunistically by daring, tricky epistemology while anticipating the best development.

Crucially, action of living entities necessitates fictional anticipatory scripts encoded in dispositional or improvisable preparedness. Feral anticipation is a causal force in a delegating (Vidunas 2019) way. Likewise, human action is based prototypically on beliefs, thus on principally presumed and fallible knowledge. That beliefs shape our conduct is an old insight of pragmatists (Peirce et al. 1935: 5.370) and others. In particular, utopias and reformative visions drive most of determined political action, for better or worse. Nietzsche (1995: Ch. 7) writes, “action requires the veil of illusion.”

I highlight two fictionalist aspects of anticipation that counter the leading contemporary paradigm of cognition based on predictive coding (Friston et al. 2016): primitive forms of anticipation look more like prejudice, superficial bias rather than objective inference; and the basic existential epistemology may have a boldly vigorous rather than a soundly careful character. In Nietzsche’s (1995) terms, living beings are life-affirming Dionysian rather than rational Apollonian. The wilder epistemological impulses are moderated by generalized natural selection.
